# FOXO3 LONGEVITY GENOTYPE AND CEREBROVASCULAR DISEASE

**DOI:** 10.1093/geroni/igad104.0778

**Published:** 2023-12-21

**Authors:** Kazuma Nakagawa

**Affiliations:** Kuakini Medical Center, Honolulu, Hawaii, United States

## Abstract

The influence of the FOXO3-longevity associated genotype on cerebrovascular disease (CVD) is little studied. There are reports of a protective effect against stroke mortality in some populations. But these findings have not been widely replicated, nor have potential mechanisms been identified. This presentation will present recent, novel insights on the relation of FOXO3 genotype to CVD. We studied a cohort of over 800 American men of Japanese ancestry from the Kuakini Honolulu Heart Program (KHHP) and Kuakini Honolulu-Asia Aging Study (KHAAS). This population-based dataset included brain autopsy data and age-adjusted prevalence of various types of CVD, including intracerebral hemorrhage (ICH) and cerebral microinfarcts (CMI). Logistic regression models, adjusted for age at death, cardiovascular risk factors, FOXO3 and APOE-ε4 genotypes, among other risk factors were utilized to determine the predictors of both outcomes. FOXO3 genotype did not directly modify risk for ICH or CMI. However, the FOXO3 longevity genotype had significant interaction (protective) effects against hypertension-associated ICH and CMI. These data suggest that the longevity-associated FOXO3 G-allele mitigates the impact of hypertension on the risk of ICH and CMI. Further research is needed in other populations.

